# Polypyrrole/Agarose Hydrogel-Based Bladder Volume Sensor with a Resistor Ladder Structure

**DOI:** 10.3390/s18072288

**Published:** 2018-07-14

**Authors:** Mi Kyung Kim, Sungwoo Lee, Inug Yoon, Geon Kook, Yeon Su Jung, Sarah S. M. Bawazir, Cesare Stefanini, Hyunjoo J. Lee

**Affiliations:** 1 School of Electrical Engineering, Korea Advanced Institute of Science and Technology, Daejeon 34141, Korea; kmkyung@kaist.ac.kr (M.K.K.); lsw9403@kaist.ac.kr (S.L.); inug.yoon@kaist.ac.kr (I.Y.); rbrrus@kaist.ac.kr (G.K.); vitadolce0@kaist.ac.kr (Y.S.J.); 2 Biomedical Engineering, Khalifa University, Abu Dhabi 127788, UAE; sarah.bawazir@kustar.ac.ae (S.S.M.B.); cesare.stefanini@kustar.ac.ae (C.S.)

**Keywords:** bladder volume, bladder monitoring, switch contact mode, resistor structure, small strain

## Abstract

Chronic monitoring of bladder activity and urine volume is essential for patients suffering from urinary dysfunctions. However, due to the anatomy and dynamics of the bladder, chronic and precise monitoring of bladder activity remains a challenge. Here, we propose a new sensing mechanism that measures the bladder volume using a resistive ladder network with contact switches. Instead of measuring the impedance between the electrode continuously, the proposed sensor provides a digitized output (‘on’ or ‘off’) when the bladder volume reaches a certain threshold value. We present simple proof-of-concept sensors which compare the discrete-mode operation to the continuous-mode operation. In addition, by using multiple pairs of this contact-mode switch in a resistor ladder structure, we demonstrate monitoring of the bladder volume in four discrete steps using an idealized balloon and an *ex vivo* pig’s bladder. We implemented the resistive ladder network using a conductive polypyrrole/agarose hydrogel composite which exhibits a Young’s modulus comparable to that of the bladder wall. Compared to the continuous-mode operation, the proposed sensing mechanism is less susceptible to drift due to material degradation and environmental factors.

## 1. Introduction

Urinary dysfunctions, which affect over 200 million people worldwide, encompass a wide spectrum of symptoms, such as voiding dysfunction, urinary incontinence (UI), and overactive bladder [1]. People suffering from urinary dysfunctions range from nocturnal enuretic children, elderly women with muscle weakness, cancer patients, and paraplegic and quadriplegic individuals with complete loss of bladder control [2,3]. Regardless of the degree of severity, loss of bladder control deteriorates the patient’s quality of life. For instance, while patients with complete loss of bladder control must employ a Foley catheter and drainage leg bag, patients with mild UI suffer from sudden involuntary leakage of urine when sneezing, laughing, and exercising [4,5]. Information on the bladder activity and urine volume through chronic monitoring would be essential for early warnings, biofeedback training, and artificial stimulation. However, due to the anatomy and complex dynamics of the bladder’s movement, chronic and precise monitoring of bladder activity remains a challenge. 

For clinical assessment of bladder functions, urodynamic testing based on fast cytometric filling and catheterization is often conducted. However, due to low patient compliance, long-term monitoring using catheterization is impractical. For patients with mild UI conditions, there are several non-invasive means to monitor the bladder, such as electric alarms [6], ultrasound monitoring [7,8], and bioelectrical impedance analysis [9,10]. Although the accuracy of these non-invasive means heavily depends on factors, such as the position of electrodes, humidity, and body temperature, these methods are attractive alternatives for ambulatory patients whom a pharmaceutical approach is not effective. For patients with complete loss of bladder control, surgical interventions are recommended as treatment. For example, an artificial urinary sphincter (e.g., AMS 800, Boston Scientific Corporation, Marlborough, MA, USA) completely closes and opens the urethra through an occlusive cuff upon the patient’s control. An implantable stimulation device (e.g., Interstim, Medtronic, Minneapolis, MN, USA) could control the sensation of urgency and empty the bladder for paraplegic and quadriplegic individuals. However, for these invasive means, occlusive cuffs exert high pressure on the urethra which causes mucosal coaptation and eventually incomplete urethral occlusion. These problems could be addressed by using an implantable bladder volume sensor that enables smart control of the timing and pressure of the stimulator [11]. 

**Figure 1:**
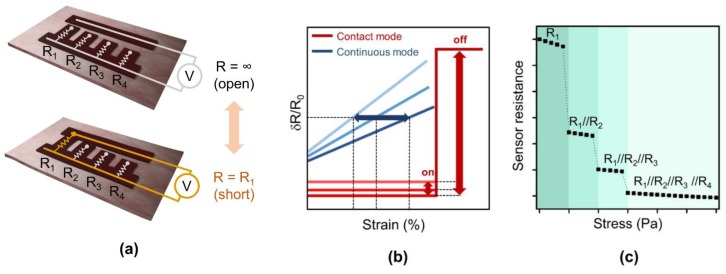
Operational principle of proposed ladder switch sensor. (**a**) Schematics of the contact-mode sensor with four pairs of electrodes before and after the distension; (**b**) Schematic illustration of resistance change of continuous strain sensor and discrete contact-mode of a single pair of electrodes over time; (**c**) Simulation results of a sensor composed of four pairs of electrodes showing the overall sensor resistance over increasing stress.

Several types of implantable volume sensors have been reported which estimate the bladder volume based of different physical and physiological parameters: neural afferent activities [12,13], pressure [14,15], distension/strain [16,17,18,19], and impedance of the bladder wall [20]. While an electroneurogram (ENG) that measures the frequency of neural activities suffers from low signal-to-noise ratio, estimation of the volume from intravesical pressure has been noted to be difficult due to the elastic nature of the bladder [21]. Measurements of bioimpedance, distension, and strain offer a simple systematic solution for wireless monitoring, but are subject to continuous drifts due to fibrotic encapsulation and natural material degradation. Due to this long-term drift, frequent sensor calibration is often required. In this work, to resolve this drift problem, we propose an implantable device that measures the distension of the bladder on discrete mode using multiple electrical switches composed of two electrodes. Instead of measuring the impedance between the electrode continuously, the proposed sensor is based on the contact mode and provides a digitized output (‘on’ or ‘off’) when the bladder volume reaches a certain threshold value. By using multiple pairs of these contact-mode switches in a resistor ladder structure, we demonstrate monitoring of the bladder volume in four discrete steps (Figure 1a). In addition, due to its small size, such a miniature implantable bladder volume sensor could serve as a new tool in animal experiments to investigate the underlying mechanism of urinary diseases and monitor the effects of new therapeutics.

## 2. Materials and Methods

### 2.1. Fabrication of Proof-Of-Concept Devices

We fabricated two proof-of-concept strain sensors to compare the performance of different operational modes: continuous and discrete. Each sensor was fabricated using conductive elastomer which consists of carbon nanotubes (CNTs) (CNT COMPANY, Incheon, Korea) and polydimethylsiloxane (PDMS) (Sewang Hitech, Gyeonggi-do, Korea). Specifically, a PDMS base and toluene were first mixed at 1:4 volume ratio and 0.3 g of CNTs were dispersed in ~7 mL of fresh toluene. Then, we mixed two solutions together for 12 h at 42 °C. The PDMS curing agent was finally added to form an 820-μm-thick CNT/PDMS composite film. For the contact-mode sensor, the CNT/PDMS composite film was cut in half, and a thin 15/80-nm layer of chrome/gold was evaporated on the contact planes to minimize contact resistance. Both sensors were then encapsulated with two 600-μm-thick PDMS layers through O_2_ plasma treatment. For resistance measurement, the sensors were electrically interfaced with two pieces of copper tape which were glued to both ends of the sensor using a silver paste (CANS, Gyeonggi-do, Korea). 

### 2.2. Fabrication of Polypyrrole/Agarose Hydrogel-Based Discrete Contact-Mode Sensor

**Figure 2:**
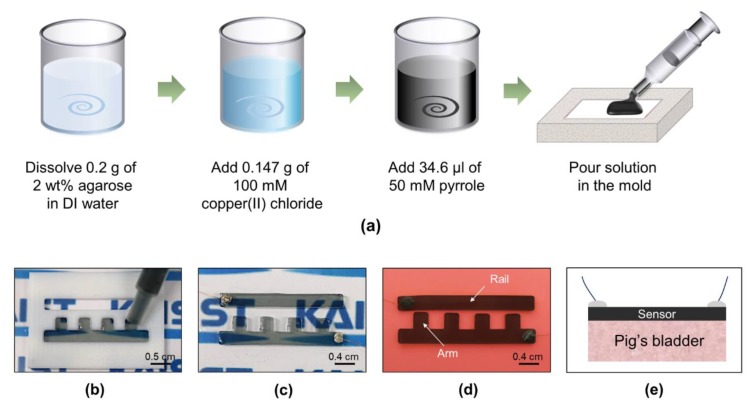
(**a**) Fabrication process of the sensor based on a polypyrrole/agarose hydrogel composite; Optical pictures of (**b**) the solution casting process; (**c**) the released sensor interfaced with wires; and (**d**) the fabricated sensor placed on a balloon; (**e**) Cross-section scheme of the sensor on the bladder.

We fabricated the proposed ladder-structured discrete contact-mode sensor using polypyrrole/agarose hydrogel composite (Figure 2). Similar to other organs, the bladder exhibits a relatively low Young’s modulus of approximately 10 kPa. It is critical for the discrete contact-mode sensor to follow the movement of the bladder closely and, thus, the mechanical property of the sensor, such as the Young’s modulus, should closely match that of the bladder. Thus, we used 2 wt% agarose (Bio-Rad, Seoul, Korea) with 50 mM pyrrole (Sigma-Aldrich, Gyeonggi-do, Korea) and 100 mM copper (II) chloride (Sigma-Aldrich, Korea). First, 0.2 g of agarose powder was dissolved in hot DI water and was cooled to 70 °C. Next, 0.147 g of copper(II) chloride as an oxidizing agent and 34.6-μL of pyrrole were sequentially added and stirred for 10 min, respectively. Finally, the mixture was cast onto a custom-designed Teflon mold using a 1-mL syringe. The sensor was released from the mold after 1 h. After interfacing the sensor with wires, the sensor was attached manually on the bladder without an adhesive layer as polypyrrole/agarose hydrogel showed sufficient adhesion to the bladder (Video S1).

### 2.3. Finite Element Simulations of Discrete Contact-Mode Sensor

To investigate the effects of various design parameters, we simulated the operation of the proposed structure using a commercial finite element simulation program (COMSOL Multiphysics^®^, Burlington, MA, USA). We modeled the bladder as a perfect sphere with a Young’s modulus of 10 kPa, bladder wall thickness of 2 mm, and a volume of 800 mL. By varying the Young’s modulus of the sensor from 0.1 kPa to 1 MPa, we examined the stress incurred on the bladder when the bladder contracted from 800 mL to 400 mL. Additionally, when the bladder volume reached 400 mL, the maximum contact gap between the rail and the smallest arm was extracted. In this work, to confirm the sensor operation, we used a constant Young’s modulus for the bladder. However, for more accurate modeling of the bladder, the viscoelastic property of the bladder wall should be reflected in the simulation [22]. In addition, since the accurate modeling of viscoelasticity of individual bladder is difficult, an initial calibration should be conducted during the surgery to determine four volume regions.

### 2.4. Measurement of Sensor Response

**Figure 3:**
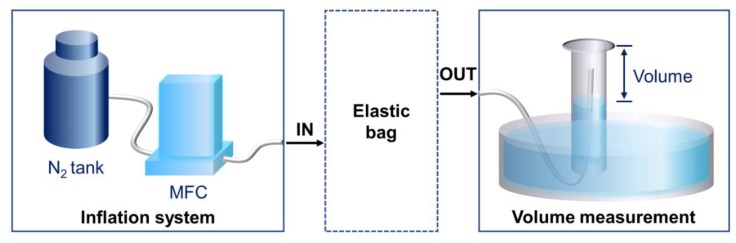
Schematics of the experimental set-up of inflation and deflation of the balloon and bladder, accompanied with volume measurement.

The fabricated sensor was characterized in terms of its electrical and mechanical properties. The conductivity of the fabricated sensor was measured using a high resistance meter (DKS-11, DASOLENG, Cheongju, Korea) while the Young’s modulus of polypyrrole/agarose hydrogel composites with different ratios was measured using a tensile testing system (MCT-2150, AND KOREA, Seoul, Korea). The change in resistance due to the change in strain, as well as the long-term cycling test of the proof-of-concept sensors, were measured using the tensile testing system and an impedance analyzer (E4990A, Keysight Technologies, Santa Rosa, CA, USA). For the fabricated contact-mode sensor, we evaluated the sensor performance by using a balloon as an ideal elastic substrate and an *ex vivo* pig’s bladder. To mimic the original bladder shape and *in vivo* experiment more closely, the balloon and *ex vivo* bladder were inflated with nitrogen (N_2_) gas at a speed of 500 mL/min using mass flow controller (MFC) to confirm the operation of our sensor. We used nitrogen gas instead of liquid because the flow rate was limited by our MFC. In the succeeding studies, to model the bladder movement more accurately and observe the operation of our sensor in a non-ideal environment, we should fill the bladder supported by other organs with liquid at a slow rate [23]. After inflation, the fabricated sensors were then attached to both a balloon and an *ex vivo* bladder. The change in resistance was monitored as nitrogen gas was slowly released at approximately 2mL/s. The amount of gas released from the balloon or the bladder was measured by measuring the gas volume in a water-filled vertical glass cylinder (Figure 3).

## 3. Results

### 3.1. Working Principle of the Discrete Contact-Mode Sensor

We propose a multi-level resistor ladder network with physical contact switches to measure the distension of the bladder (Figure 1a). The sensor is composed of two electrodes which are initially separated by a narrow gap. When the substrate underneath the sensor shrinks beyond a certain threshold, the two electrodes are pulled closer to each other and eventually form an electrical contact (i.e., contact switch). As a result, the impedance of the sensor abruptly changes from infinite (open) to ideally zero (short) in a binary manner. By using an additional pair of these electrodes, but with different gap distances and connecting them in parallel, an additional range of volume can be defined (Figure 1c). For instance, four electrode pairs with different gaps connected in parallel would allow for the estimation of four distinctive volume ranges. As the bladder further contracts, the number of electrodes in contact sequentially increases, and the overall resistance of the sensor decreases in a discrete manner. If the resistance of each electrode is the same, the sensor resistance decreases by 1/*n* where *n* is the number of electrodes in contact. This structure resembles a multi-level resistor ladder network used in conventional electrical engineering [24]. By attaching this multi-level resistor ladder on the outer wall of the bladder, we aim to measure the distension of the bladder and estimate the volume.

### 3.2. Comparison of a Continuous Strain Sensor and a Discrete Contact-Mode Sensor

**Figure 4:**
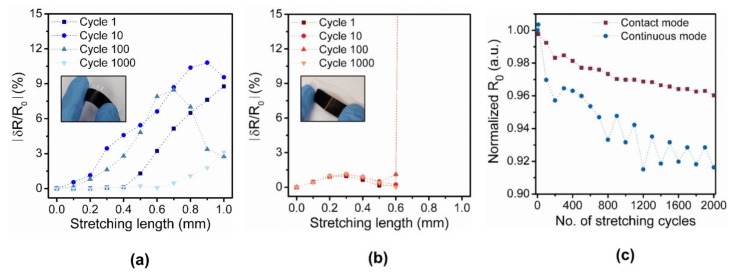
Sensor performance of continuous strain sensor and contact-mode sensor. (**a**) Measured fractional resistance change of the continuous strain sensor over 1000 cycles (inset: optical picture of the proof-of-concept continuous strain sensor composed of CNT/PDMS composite); (**b**) Measured fractional resistance change of the contact-mode sensor over 1000 cycles (inset: optical picture of proof-of-concept contact-mode sensor with one pair of electrodes; (**c**) Normalized initial resistance, R_0_, of two proof-of-concept sensors over 2000 stretching cycles.

To confirm the working principle and advantage of the contact-mode sensor, we measured the long-term stability of two proof-of-concept devices: a continuous strain sensor and a contact-mode sensor with one pair of electrodes (Figure 4a,b). Fractional resistance change *(δR/R*_0_) of both sensors were measured while both sensors were stretched from 0 to 1 mm over 1000 stretch/relax cycles, where *R*_0_ is the initial resistance value of the sensor. For the continuous strain sensor, the fractional resistance change was small and varied extensively between each cycle (Figure 4a). Due to the large variation, estimating the strain from the measurement was difficult. On the contrary, for the contact-mode sensor with one pair of electrodes, a single binary detection of strain smaller or larger than a threshold strain was possible (Figure 4b). For the strain larger than 0.6 mm, the fractional resistance changed abruptly from approximately 1 (short) to a large number (ideally open). Although the resolution of this contact-mode sensor was low (i.e., binary), the cycling test showed an excellent reproducibility (threshold value of ~0.6 mm) due to the large difference in resistance value. Moreover, the change in the initial resistance value over 1000 cycles was more stable for the contact-mode sensor because the stress applied on the sensor, which induces micro-cracks, was smaller for the contact-mode sensor due to the gap in the middle (Figure 4c). Thus, due to the binary operation, the contact-mode sensor was less susceptible to environmental changes (Figure 1b). To achieve a higher resolution, we can use multiple binary sensors connected in parallel.

### 3.3. Design and Optimization of a Discrete Contact-Mode Sensor

**Figure 5:**
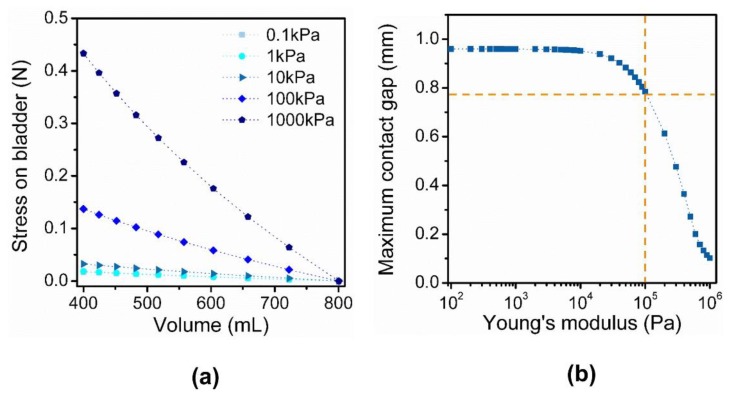
Finite element simulation results of the sensor operation. (**a**) Stress imposed on the bladder by the sensor with different Young’s modulus; (**b**) Maximum gap distance between two electrodes to form an electric contact when the volume changes from 800 mL to 400 mL.

**Figure 6:**
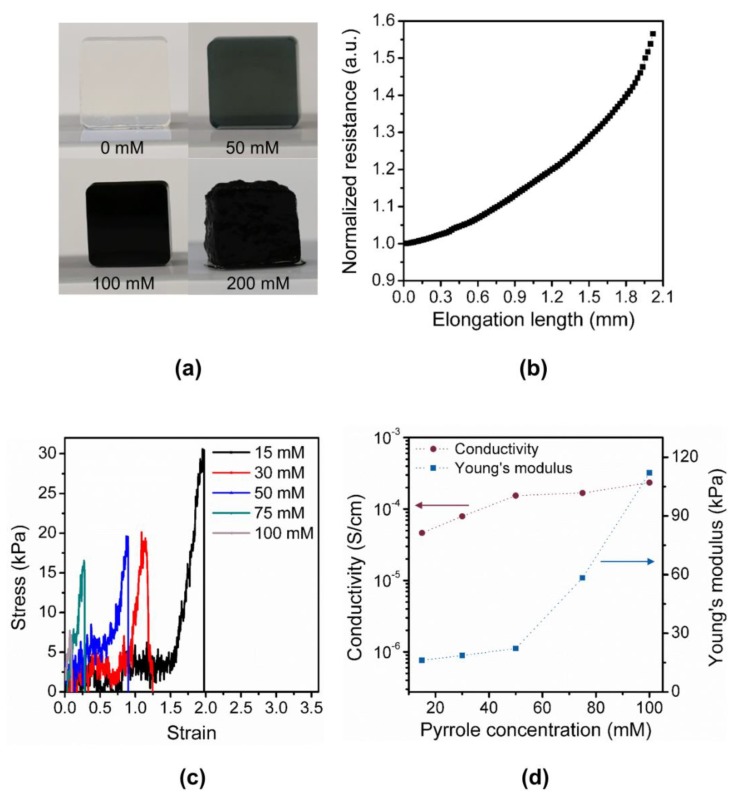
Material properties of the contact-mode sensor. (**a**) Optical pictures of the polypyrrole/agarose hydrogel block with different concentrations of pyrrole; (**b**) Normalized resistance of polypyrrole/agarose hydrogel composite over different elongation lengths; (**c**) Stress measurement of a 1-mm-thick polypyrrole/agarose hydrogel composite with different pyrrole concentrations over different strain values; (**d**) Measured conductivity and Young’s modulus over different concentrations of pyrrole.

We implemented the multi-level resistor ladder network using a soft and conductive hydrogel. The multiple pairs of electrodes connected in parallel was implemented as two long rails with four arms attached on one of the rails (Figure 2d). Different gap distances between the electrodes were achieved by varying the lengths of the four arms. As the elastic substrate distends, the arms formed the contact with the opposite rail sequentially according to the gap distances. As predicted, distinctive changes in resistance over the four contact regions were observed (Figure 1c). For the contact-mode sensor, it is essential for the sensor to move in response to the distension of the substrate and also not to impose stress on the bladder. Thus, the Young’s modulus of the sensor material in comparison to that of bladder wall is a critical design parameter. The simulation of sensor operation showed that the stress imposed by the sensor on the bladder was minimal for a Young’s modulus between 10 kPa to 100 kPa (Figure 5a). For the sensor with a Young’s modulus larger than that of a bladder wall (~10 kPa), stress imposed on the bladder was high, especially when the contraction of the bladder was high (i.e., low bladder volume). Thus, for our design, we adjusted the concentrations of the polypyrrole/agarose hydrogel composite to achieve a Young’s modulus closer to that of the bladder wall (Figure 6a). As the pyrrole concentration increased, both the Young’s modulus and conductivity of the composite increased (Figure 6c,d).

In addition to the mechanical property of the material, geometric parameters are also important in determining the sensor performance. Specifically, the dynamic range of the sensor is determined by the gap distance of the first and the fourth pairs of electrodes, which set the minimum and maximum detectable volume range. If the bladder is assumed to shrink from 800 mL to 400 mL, there exists a maximum gap distance for the fourth pair of electrode. If the gap distance of the fourth pair is larger than this value, the fourth electrode pair will not form a contact despite the full contraction. Assuming a perfect sphere, a Young’s modulus of 10 kPa for the bladder, and the change of the bladder volume from 800 mL to 400 mL, we simulated the sensor operation to estimate the maximum gap distance of the fourth pair for different Young’s modulus of the sensor (Figure 5b). As the Young’s modulus of the sensor decreased, the maximum gap distance required to establish a full contact increased and saturated to approximately 0.78 mm when the Young’s modulus was 100 kPa. In addition, simulation results showed that the maximum gap distance was not a function of the length of the fourth arm. Thus, for easier handling of the sensor, we designed the smallest arm length to be 3 mm and the gap distance to be smaller than 0.7 mm. In addition, we simulated the operation of the sensor over the typical bladder volume from 600 mL to 100 mL and used the measured elastic modulus, conductivity of the sensor, and contact gap (Figure S1a,b). The sensor operated as predicted over the new range; the arm and the opposite rail formed a contact while the pressure between the contact increased as the bladder contracted (Figure S1c–f). Furthermore, the stress incurred on the bladder and the current flowing through the sensor were also simulated (Figure S2). As the bladder volume contracted below 243 mL, the longest arm was distorted in the simulation (Figure S1g). This result shows that the dynamic range of our sensor is determined not only by the smallest arm but also by the longest arm. To design the sensor to operate below 243 mL, the material property or the length of the longest arm should be adjusted.

### 3.4. Balloon and Ex Vivo Experimental Results

**Figure 7:**
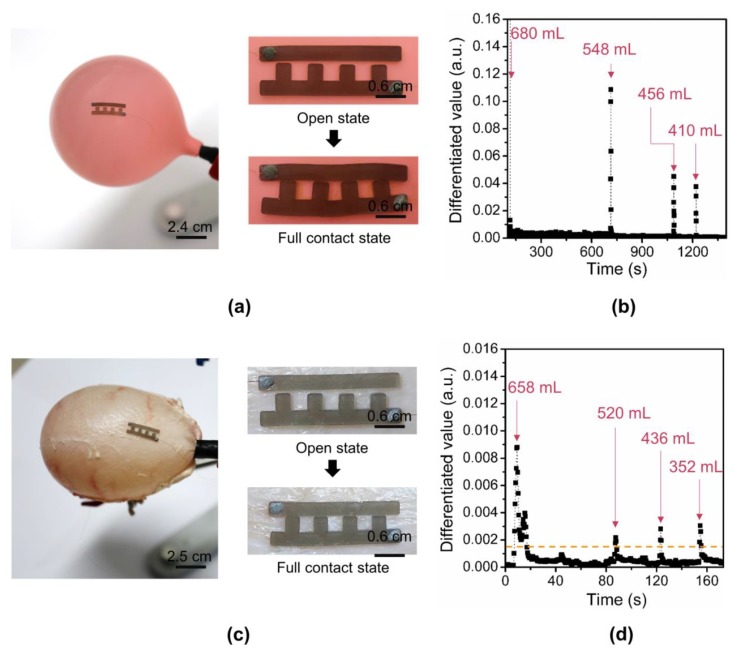
Demonstration of sensor operation on the balloon and *ex vivo* pig’s bladder: (**a**) Photo of the sensor attached to an ideal balloon and the zoomed-in photos of the sensors in all-open and all-closed states; (**b**) Absolute differentiated value of the sensor attached to a balloon; (**c**) Photo of the sensor attached to an *ex vivo* pig’s bladder and the zoomed-in photos of the sensors in all-open and all-closed states; (**d**) Absolute differentiated value of the sensor attached to an *ex vivo* pig’s bladder.

To confirm the operation of the proposed sensor structure, we first affixed the polypyrrole/agarose hydrogel sensor with four arms on a balloon (Figure 7a). The maximum gap distance of the fourth pair of the electrodes was set to 0.5 mm. The balloon offers an ideal test substrate with high substrate impedance and spherical distension. While the 822-mL filled balloon was deflated by releasing the nitrogen slowly, the volume of the released air, as well as the sensor resistance, were measured and recorded. The first arm formed a contact with the opposite rail at 680 mL, and the consecutive arms formed a contact sequentially at 548 mL, 456 mL, and 410 mL, respectively (Figure 7b). The output of the sensor was obtained by first normalizing the sensor resistance to the initial value and differentiating the resistance after normalization. As predicted, the absolute value of differentiated resistance value abruptly increased as the number of contacts in sensor increased. We used differential resistance as the output because the arms in contact with the opposite rail were gradually compressed as the volume decreased and thus the resistance of the arm decreased (Figure 6b and Figure S3a).

The functionality of the sensor was also confirmed by affixing it on an *ex vivo* pig’s bladder (Figure 7c). Unlike the ideal balloon, the pig bladder is not a perfect sphere and contracts and expands in anisotropic directions. Thus, through various trials, we placed the sensor near the bladder neck with two rails separated with the maximum contact distance of 0.5 mm. As the bladder was emptied from 818 to 352 mL, the sensor resistance decreased as the number of electrodes in contact increased (Figure S3b). Similar to that of the balloon, as the number of contacts increased, the value of the absolute differentiated value showed an abrupt increase. The first arm formed a contact with the opposite rail at 658 mL, and contacts were gradually formed at 520 mL, 436 mL, and 352 mL, respectively (Figure 7d). Based on the baseline of ~0.0015, the bladder volumes of four ranges were distinguished.

## 4. Discussion

In this work, we demonstrated the feasibility of discrete contact-mode sensing to estimate the volume of both balloon and *ex vivo* pig’s bladder. As demonstrated by the proof-of-concept devices, the advantage of the proposed sensing method is less susceptibility to long-term drift, which was achieved in a trade-off to the sensor resolution. While the dynamic range of the sensor is adjusted by the maximum gap distance, the resolution of the proposed structure is a function of the number of arms with different lengths. Thus, the sensor resolution can be improved by increasing the number of arms. To implement a practical implantable sensor based on the proposed resistor ladder structure, additional features, such as calibration, encapsulation, and interface circuits, must be considered. First, a calibration process which determines the optimal position of the sensor on the bladder and four distinctive volume regions is required due to anisotropic contraction and expansion and the viscoelastic nature of the bladder. Second, the electrical insulation between the sensor and the bladder wall is required to minimize the change in resistance over the discrete region. Additionally, the lifetime of the sensor and the sensitivity will be enhanced by encapsulation. However, when the sensor is encapsulated, the mechanical property of the sensor is modified and, thus, the threshold values for each volume’s regions should be re-evaluated. Various encapsulation methods have been proposed, including hermetic sealing and surface modification. To protect the entire implanted device from body fluids, hermetic packaging with biocompatible materials, such as Parylene and PDMS, are desired [25,26]. In addition, drug coating of the surface could enhance the biocompatibility and reduce the biological response, such as biofouling of the implanted device [27]. Such encapsulation would not only prevent impedance changes due to biofouling, but also provide electrical insulation to the bladder wall. Moreover, the change in resistance over the discrete region can be minimized by adjusting the compositions of the polypyrrole/agarose hydrogel, such as doping, anthraquinone-2-sulfonic acid (ASA) and 5-sulfosalicylic acid (SSA) [28]. Lastly, the proposed sensor requires a read-out circuit which either directly interfaces with the stimulation device [29] or transmits the data wirelessly [30]. For practical use of the sensor after implantation, more investigations are required to achieve robust operation, such as calibration and read out schemes in the succeeding development. During the implantation, the sensor should be initially calibrated to find the optimal sensor position and to find the volume range. In addition, to compensate for geometric variations of the bladder during the filling and voiding cycle due to body position and neighboring organ position, an additional number of sensors could be used. Lastly, a read-out system must be designed and implemented; one possible solution for data transfer for our sensor is a wireless system attached to the skin that transmits power and receives data over a short distance [31].

In summary, we have proposed and demonstrated a new sensing mechanism of bladder volume using a resistor ladder structure. Contrary to widely-used strain sensors that measure stretchability in the analog mode, the proposed sensor measures the volume through the discrete contact mode. While analog sensors suffer from long-term drift due to undesirable effects, such as material degradation and environmental effects, sensors with discrete outputs are less susceptible to long-term drift. By using a soft and compressive polypyrrole/agarose hydrogel composite with a Young’s modulus comparable to that of the bladder, we have demonstrated the feasibility of a contact-mode sensor to monitor the volume of both a balloon and *ex vivo* bladder. Based on the advantage of high reliability, the proposed discrete contact mode sensing is a promising alternative method to measure volume and small changes in strain.

## Supplementary Materials

The following are available online at http://www.mdpi.com/1424-8220/18/7/2288/s1, Figure S1: Finite element simulation results of the sensor operation, Figure S2: Finite element simulation results of the sensor operation as the bladder contracted from 600 ml to 243 ml, Figure S3: Normalized resistance of the sensor attached on the balloon and bladder, Video S1: Sensor on the bladder without an adhesive layer.
