# Supplementary material for: Polypyrrole/Agarose Hydrogel-Based Bladder Volume Sensor with a Resistor Ladder Structure
Source: Sensors (Basel). 2018 Jul 14;18(7):2288. doi: 10.3390/s18072288 (PMC6069483; doi:10.3390/s18072288)
Supplement: Supplementary file 1 [file sensors-18-02288-s001.pdf]

Supplementary Information

Polypyrrole/Agarose Hydrogel-based Bladder Volume Sensor with a Resistor Ladder Structure

Mi Kyung Kim [1], Sungwoo Lee [1], Inug Yoon [1], Geon Kook [1], Yeon Su Jung [1], Sarah S. M. Bawazir [2], Cesare Stefanini [2], and Hyunjoo J. Lee [1,*]

[1] School of Electrical Engineering, Korea Advanced Institute of Science and Technology, Daejeon 34141, Republic of Korea; kmkyung@kaist.ac.kr
[2] Biomedical Engineering, Khalifa University, Abu Dhabi 127788, United Arab Emirates
* Correspondence: hyunjoo.lee@kaist.ac.kr; Tel.: +82-42-350-7436

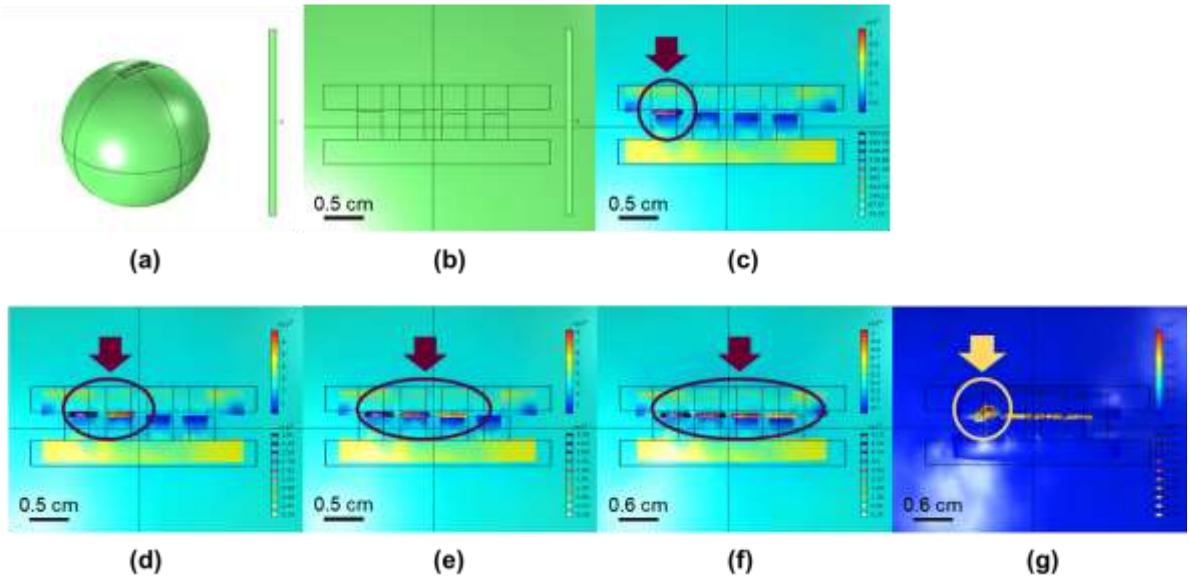

Figure S1. Finite element simulation results of the sensor operation: (a) Bladder model. (b) Original state of the sensor on the bladder. (c)-(f) Movement of the sensor as the bladder volume contracted to: (c) 466 ml, (d) 394 ml, (e) 364 ml, and (f) 315 ml. Circles and arrows indicate the contacts formed between the rail and the arm. (g) Abnormal operation of the sensor when the volume decreased below 243 ml due to the high stress incurred on the longest arm.

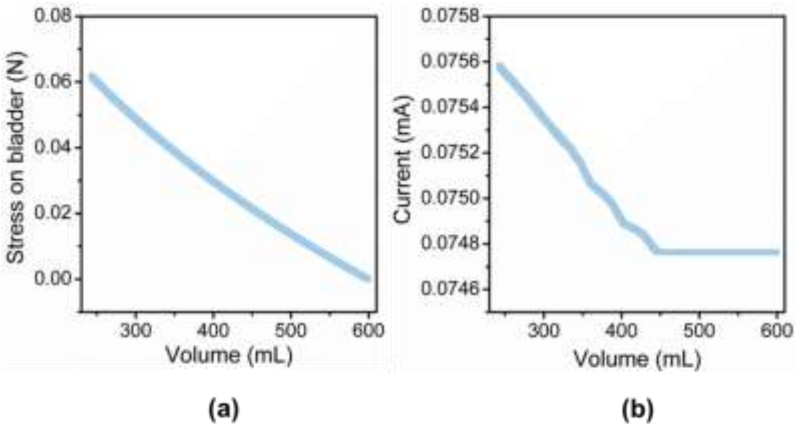

Figure S2. Finite element simulation results of the sensor operation as the bladder contracted from 600 ml to 243 ml: (a) Stress imposed by the sensor on the bladder. (b) Current through the sensor.

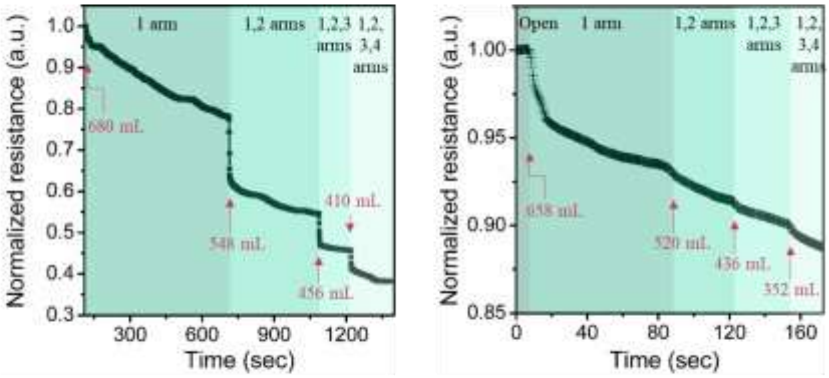

(a) (b)

Figure S3. (a) Normalized resistance of the sensor attached on the balloon. (b) Normalized resistance of the sensor attached on the bladder.

bladder_no adhesive.mp4

bladder_no adhesive2.MOV

Video S1. Sensor on the bladder without an adhesive layer.
